# The Memphis Journal of the Medical Sciences, for February

**Published:** 1890-03

**Authors:** 


THE MEMPHIS JOURNAL OF THE MEDICAL SCIENCES, for February, 1890, is before us.

The leading article, "Homœopathy, a Persistent Medical Superstition," by R. G. Eccles, M. D., is exceedingly interesting, and arrests attention at once.

It compares Homœopathy to the superstitions of the ancients and of savages.

It recalls the doctrine of signatures, and declares that the law of the similars and the law of the contraries were both ancient superstitions, and that

"in 1796 a German physician, named Hahnemann, began a crusade for the restoration of the old doctrine with all its exclusiveness."

The author declares that "there never has been any organized body of physicians calling themselves allopaths, nor would such a title be true except in rare instances." Further, he says, that most of the physicians of our school are ashamed of Hahnemann's "attempt to prove that all chronic maladies are but masked manifestations of the itch. Idiocy, gout, cancer, insanity, hysteria, and debility, he taught, were all due to patients catching the itch." Before the acarus scabiei was discovered they were just as certain he was right in that as they now are of his main doctrine.

Next follows an argument upon the potency question.

The author has been looking over some catalogue of remedies, for he says: "To think that men claiming the degree of doctor of medicine can so far get rid of their senses as to believe that a beam of sunlight diluted with sugar of milk thousands of times can be of any earthly use in sickness passes comprehension. To see them adminster the ten-millionth part of a moonbeam, or of a red, yellow, green, or blue light so diluted is to make us pity frail humanity."

Then follows a list of the most abominable things that can be conceived of, that are being used in medicine, and declares that they far exceed the efforts of Shakespeare's imagination in his description of the hell-broth of Macbeth's witches.

We suggest to any of our readers who have the leisure that they would have an instructive and highly entertaining subject for thought if they would read in full this clever article, and then turn to the February No. of *Harper's Magazine*, and read Mark Twain's article entitled "A Majestic Literary Fossil."

We can hardly predict *what* would be the effect upon the mind of the average medical man of our school by reading these two articles.

Upon the laymen the effect, we should think, would be to deepen the distrust and contempt with which all medical men are regarded by a large number of people. Well, that is because they don't understand the facts in the case.

There is an inner history to the very truthful statements of the article now in review which the author evidently does not know of. We find ourselves too limited in space and time to make any rejoinder. We can only say that if the writer will take some moderate potency of some well-known drug like Aconite, Belladonna, or Nux vomica, and try it according to the law of the similars he will be surprised at the results. W. M. J.

